# Research on the Tournament Incentive Mechanism of the Safety Behavior for Construction Workers: Considering Multiple Heterogeneity

**DOI:** 10.3389/fpsyg.2021.796295

**Published:** 2021-12-13

**Authors:** Liuyang Ji, Wenyao Liu, Yifan Zhang

**Affiliations:** School of Management, Jiangsu University, Zhenjiang, China

**Keywords:** construction workers, safety behavior, tournament incentive mechanism, multiple heterogeneity, risk appetite

## Abstract

The unsafe behavior of construction workers is one of the most important and direct causes of safety accidents. Managers usually develop effective incentives aimed at regulating worker safety behavior. Due to the large number of workers in construction projects, there are multiple differences in fairness preference, risk preference and ability level, which will lead to the complex effect of the traditional mechanism to regulate workers’ safety behavior. In order to improve the effectiveness of incentive measures for worker safety behavior, this paper takes into account the multiple differences of individual workers’ fairness preference, risk preference and ability level, based on the tournament mechanism to construct a competition incentive model. By designing a tournament reward and salary distribution for heterogeneous workers, the occurrence of unsafe behaviors can be reduced. The study found that in terms of the optimal level of safety investment, workers with risk aversion attitude generally invest higher than that of workers with risk preference, no matter whether they have a strong fairness preference or not; In terms of the distribution of tournament rewards, workers with a risk aversion attitude and a higher level of fairness preference need to be given higher incentives.

## Introduction

The engineering construction process usually faces the complex challenges of the construction environment, technology and on-site management, making it more prone to safety accidents than other industries (He et al., 2020). In China, in the first half of 2018, there were 1,732 safety accidents and 1,752 deaths in the construction industry, an increase of 7.8 and 1.4%, respectively (Han et al., 2019b). In the United States, there were more than 60,000 construction worker injuries in the construction industry in 2018, which was 32.6% higher than the average for all industries (Han et al., 2019a). The construction industry has become one of the most dangerous industries, and the situation of construction safety is very severe. From the statistics on the frequency of fatal construction accidents published by the Occupational Safety and Health Administration (OSHA), the top five accidents (falling accident, electric shock accident, object strike, mechanical injury and collapse accident) are directly related to unsafe behavior of workers (Choe et al., 2016). Similarly, Oswald et al. (2018) proposed that 88% of accidents in construction projects involve unsafe behavior.

In order to effectively control and regulate the unsafe behavior of workers, managers and safety practitioners carry out a large number of interventions in the workplace, such as safety training, safety communication, safety rules and procedures, incentive measures, etc., aiming at improving workers’ safety performance (Di Tecco et al., 2017; Wang et al., 2017; Kim et al., 2019). Among them, incentive measures are a proactive method commonly adopted by management. Financial incentives including money or prizes and non-financial incentives including evaluation feedback are all helpful to regulate workers’ safety behaviors (Guo et al., 2018). However, for the construction team, the incentive problem in the construction phase is faced with more complex scenarios, and many factors such as external environment and institutional conditions, individual attributes, and dynamic interaction of multiple agents may affect the incentive effect. Among them, the existence of multiple heterogeneous attributes of workers has brought challenges to the formulation of incentive measures, which may make the incentive effect far from expectations.

First of all, the fairness preference heterogeneity of workers has a significant impact on the incentive effect of individuals. Fairness preference refers to workers’ preference for fair income distribution, which is usually manifested in jealousy when workers’ income is lower than others, resulting in negative utility. In fact, different agents have different fairness preferences. For example, some are selfish and jealous, and some emphasize fairness and reciprocity. A large number of scholars have studied the influence of fairness preference on incentive structure and incentive effect. For example, Grund and Sliwka (2005) found that fairness preference of employees will reduce the intensity of rewards and the incentive effect; Dubey et al. (2013) separately studied the influence of jealousy and pride on incentive structure and incentive effect; Gill and Stone (2015) discussed the interaction between fairness preference and self-worth in competition. Therefore, considering the heterogeneous characteristics of workers’ fairness preferences is very necessary for the study of incentive mechanisms.

Secondly, the risk attitude of workers has a significant impact on the level of safety input (Kimbrough and Componation, 2009). Risk psychology has been studied by scholars for many years, but there is still not much practical guidance in the workplace. Because risk attitude has a significant impact on all factors of risk treatment, the impact of workers’ risk attitude on construction safety should be fully considered during the project. Workers with different risk attitudes take different action choices when faced with possible risks or uncertain factors caused by information asymmetry. For a high-risk industry such as project construction, how to adjust incentive measures for workers with different risk attitudes is of great practical significance.

In addition, differences in the abilities of individuals will also affect the incentive effect on workers’ safety behavior. Due to the different learning abilities of individual workers and the mastery of knowledge system in safe operation, the time and energy spent by each worker in safety investment will vary to a large extent (Gurtler and Krakel, 2010). For example, highly educated workers pay more attention to the importance of safety investment and work harder to learn safety regulations, reducing the occurrence of unsafe behaviors; experienced workers are more likely to avoid unsafe behaviors than workers with less work experience, etc. Therefore, in order to reduce the unfairness of asymmetric competition, we need to consider the formulation of incentives for workers of different ability.

As a new branch of the reward distribution system, the tournament mechanism is a very effective incentive method. The concept of tournament incentives originated from corporate governance and was first proposed by Lazear and Rosen (1981). It is actually a compensation plan for multiple agents. It is actually a compensation scheme for multiple entities, which is paid according to the ranking of individuals or teams in the organization. Higher performance means higher salaries for subjects with higher rankings. Among them, the salary gap between the higher-ranked and lower-ranked entities reflects the incentive intensity of the tournament mechanism (Zhang et al., 2017).

At present, scholars try to study the potential application of tournament incentive in various fields, such as sports competitions, the promotion of large-scale corporate executives the promotion of large-scale corporate executives and other issues. For example, a study by Kini and Williams (2012) found that tournament incentives would stimulate executives to work harder in corporate governance, thereby increasing their chances to the position of CEO. Altmann et al. (2012) analyzed the incentive effect of the multi-stage tournament incentive mechanism and found that wage differences can improve the work level of agents. Coles et al. (2018) found that the salary gap of tournament incentives is positively correlated with output performance and corporate risk. Huang et al. (2019) suggest that industry tournament incentives can increase the market revenue of products by motivating CEOs. The above-mentioned scholars have proved that tournament incentives have a positive effect on solving the problem of multi-agent incentives. Therefore, this paper takes the tournament as an incentive mechanism for workers’ safety performance to study its influence on the unsafe behaviors in team work.

However, the current tournament mechanism must also consider some limitations and shortcomings. On the one hand, there is little literature on the impact of tournament incentives on construction project management, especially for the safety behavior performance of workers. On the other hand, the existing tournament incentive model only analyzes the impact of the pay gap on the input level of employees, and does not propose a specific salary distribution plan for heterogeneous workers. These limitations make it difficult to apply the tournament mechanism to the safety behavior incentives of construction workers in multiple heterogeneous situations. Therefore, in order to solve these problems, this research attempts to design a tournament reward distribution scheme based on the ranking of the safety performance and the degree of individual heterogeneity, to reduce unsafe behavior of workers and improve the safety performance of construction projects.

The main contributions of this article are: (1) Considering heterogeneity of the fairness preference, risk preference and abilities of the construction workers, making the motivated agents more realistic. (2) According to the characteristics of heterogeneity of construction workers, the tournament incentive mechanism was introduced into the incentive measures for workers’ safety behavior, and incentive measures were improved. This research will find a new solution for the management and control of unsafe behaviors of workers with multiple heterogeneous characteristics, and provide a reference for formulating distribution plans for controlling the behavior.

## Basic Hypotheses

In order to comprehensively analyze the competition incentive model, according to the existing relevant literature and theoretical basis, we put forward the following hypotheses.

Hypothesis 1: Two construction workers in a team participated in the tournament. The work among construction workers is independent, and their work does not affect each other. This study does not consider the help and sabotage behavior among workers. At the same time, Construction workers pay attention to their incentive income and compare it with each other. Among them, *e*_*i*_(*i* = 1,2) is the construction worker’s safety investment level. Larger *e*_*i*_ means construction workers put more effort into safe construction.

Hypothesis 2: *c*(*e*_*i*_) represents the safety input cost of construction workers. Assuming that the input cost is a convex function, it indicates that when the safety input increases, the marginal cost of the safety input increases, that is *c*(*e*_*i*_)′ > 0, *c*(*e*_*i*_)″ > 0. Therefore, the input cost of construction worker *i* is:

(1) $$c\,\left(e_{i}\right)=c\,e_{i}^{2}$$

In the formula, *c* is a constant.

Hypothesis 3: The safety performance score of construction workers π_*i*_. When designing the safety performance salary distribution of the championship, the team needs to rank the safety performance evaluation scores of two construction workers. We set the safety output performance of each worker, that is, the safety performance assessment score is related to the safety input level of construction workers and the influence degree of uncertain factors (Griffin and Neal, 2000). It can be expressed as:

(2) $$\pi_{i}=e_{i}+\varepsilon$$

where ε denotes a stochastic variable, which is subjected to a normal distribution. That is ε ∈ (0,σ^2^), representing external uncertainties. The probability that construction worker *i* ranks first is *P*_*i*_, and 1−*P*_*i*_ is the probability that construction worker *i* ranks second. According to the LR reward model (Grund and Sliwka, 2005), it can be expressed as:

(3) $$P_{i}=prob\left(\pi_{1}>\pi_{2}\right)=prob\left(e_{1}+\varepsilon_{1}>e_{2}+\varepsilon_{2}\right)=prob\left(\varepsilon_{1}-\varepsilon_{2}>e_{2}-e_{1}\right)=G\left(e_{2}-e_{1}\right)$$

Where *G*(*e*_2_−*e*_1_) and *g*(*e*_2_−*e*_1_) are respectively, the cumulative distribution function and density function of *c*(*e*_*i*_), Where *c*(*e*_*i*_) is the safety input cost function of construction workers. When the safety input level of workers is the same (*e*_1_ = *e*_2_), $G\,\left(e_{2}-e_{1}\right)=G\,\left(0\right)=\frac{1}{2}$, $g\,\left(0\right)=\frac{1}{2\,\sigma \,\sqrt{\pi }}=\frac{1}{2\,\theta }$, where $\theta =\sigma \,\sqrt{\pi }$.

Hypothesis 4: The team manager distributes the incentive reward according to the ranking order of the safety performance evaluation scores. *W*_*H*_ is the income paid to the first-ranked worker, *W*_*L*_ is the income paid to the second-ranked worker, and Δ*W* is the payment difference.

Hypothesis 5: The output utility function of workers with heterogeneous ability. We use the worker’s cost coefficient of safety input to describe the heterogeneity of worker abilities (Halisah et al., 2021; Niu et al., 2021). When α_*i*_ > 1, it means that when the safety investment is the same, the worker *i* needs to pay more, that is, the ability of worker *i* is lower.

(4) $$c\,\left(e_{i}\right)=c\,\alpha_{i}\,e_{i}^{2}$$

Hypothesis 6: According to the previous analysis of fairness preference, during the implementation of incentives, construction workers will care about whether the results are fair (Mueller, 2020; Wu et al., 2020; Yan et al., 2020). In the model based on fairness preference, ∂ represents the pride preference of workers when they win rewards, and δ is the jealous preference when they lose, 0 < ∂ < 1, 0 < δ < 1. When ∂ and δ are equal to 0, it indicates that the construction worker has no fair preference.

Hypothesis 7: λ_*i*_ represents the risk attitude coefficient of worker *i*, λ_*i*_ > 0. When 0 < λ_*i*_ < 1, it means that the risk attitude of worker *i* is preferred, so that the worker has a fluke and thinks that he can still get a better income without investing too much in safety behavior; When λ_*i*_ = 1, it means that the worker’s risk attitude is neutral; When λ_*i*_ > 1, it means that the worker’s attitude is to avoid risks. In addition, when the level of safety investment *e*_*i*_ is the same, the investment cost of employees *c*(*e*_*i*_) with risk preference is high. Then the relationship between the safety input cost of construction workers and after adding the risk heterogeneity is:

(5) $$c\,\left(e_{i}\right)=\frac{c\,\alpha_{i}\,e_{i}^{2}}{\lambda_{i}}$$

Hypothesis 8: For workers, there is also a restriction on participation. Each worker has a retention effect. The reward given by the manager must ensure that the worker is willing to stay in his position and make safe investment. The expected utility of the position must be greater than or equal to the reserved utility of his own, otherwise, he is likely to find a way to change positions or switch jobs. Suppose that for construction worker *^i^*, his retention utility is *U*.

In this chapter, we combed the relevant literature and theories, put forward a series of assumptions as the basis, and paved the way for the later model construction.

## Tournament Incentive Model Based on Heterogeneous Characteristics

### Model Establishment

(1) The expected utility of the worker ($E\,U_{i}^{H}$): According to Hypothesis 2, the net benefit of construction workers (*U*_*i*_) is determined by the income paid by the manager (*W*_*i*_), the effect of the fairness preference and the output of hard behavior *c*(*e*_*i*_). When construction workers rank first, workers will be proud of their victory. Therefore, the net benefit (*U*_*i*_) increases the positive effect of fairness preference. At this time, the net benefit of construction workers is:

(6) $$U_{i}^{H}=W_{H}+\partial \Delta \,W-c\,\left(e_{i}\right)$$

When the construction workers ranked second, the net benefit (*U*_*i*_) reduced the negative effect of fairness preference. At this time, the net benefit of construction workers is:

(7) $$U_{i}^{L}=W_{L}-\delta \,\Delta \,W-c\,\left(e_{i}\right)$$

Thus, the expected utility of the worker *i* is:

(8) $$\begin{array}{c} E\,U_{i}=P_{i}\,U_{i}^{H}+\left(1-P_{i}\right)\,U_{i}^{L}\\ =P_{i}\,\left(1+\partial +\delta \right)\,\Delta \,W+W_{L}-\delta \,\Delta \,W-c\,\left(e_{i}\right) \end{array}$$

(2) The manager’s net benefit (*EU*_*i*_):

According to Hypothesis 4, the safety output performance of construction workers is π_*i*_, and the expenditure of managers is *W*_*i*_. Since managers are risk-neutral, the expected net income of managers is:

(9) $$E\,U=\pi_{i}-W_{i}=\pi_{i}-e_{i}-\varepsilon$$

(3) Competition incentive model: The incentive for construction workers is equivalent to solving the following problems:

(10) $$\max E\,U=\pi_{i}-W_{i}=\pi_{i}-e_{i}-\varepsilon$$

(11) $$E\,U_{i}^{H}=P_{i}\,\left(1+\partial +\delta \right)\,\Delta \,W+W_{L}-\delta \,\Delta \,W-\frac{c\,\alpha_{i}\,e_{i}^{2}}{\lambda_{i}}\geq U$$

(12) $$\max E\,U_{i}=P_{i}\,\left(1+\partial +\delta \right)\,\Delta \,W+W_{L}-\delta \,\Delta \,W-\frac{c\,\alpha_{i}\,e_{i}^{2}}{\lambda_{i}}$$

According to the incentive model solution method, the optimal safety investment level of construction worker *i* is calculated:

(13) $$e_{i}^{*}=\frac{h\,\left(e_{i}-e_{j}\right)\,\left(1+\partial +\delta \right)\,\Delta \,W\,\lambda_{i}}{\alpha_{i}}$$

From the proof of Equation (13), we can know (1) The optimal safety input level *e*_*i*_ of construction workers is positively correlated with the pride preference ∂ of workers when they win the reward, jealousy preference δ of workers when they lose the reward, compensation gap Δ*W* and risk preference level λ_*i*_, and negatively correlated with the cost coefficient of safety input α ; (2) In addition, $e_{i}^{*}$ increases with the increase of incentive compensation gap Δ*W*. That is, the safety level of worker input has nothing to do with the absolute size of the bonus itself. That is, the safety level of worker input has nothing to do with the absolute size of the bonus itself. And it has to do with the difference between bonuses. The greater the difference, the higher the level of safety investment. Based on the principle of the tournament, the total amount is reduced, but the difference remains unchanged, and the desired incentive effect can also be achieved. Therefore, tournament incentives can achieve the expected incentive effect by paying different incentives to different workers according to their ranking in the tournament; (3) The optimal safety input level $e_{i}^{*}$ decreases with the increase of safety input cost coefficient α_*i*_. When α_*i*_ increases gradually, the difference between worker *i* and worker *j* increased. At this time, the safety input level of worker *i* will be significantly reduced, the winning probability of worker *j* will increase, thus the safety input level of worker *j* will also decrease, and the probability of accidents caused by unsafe behavior will greatly increase. Therefore, for team managers, how to allocate personnel and resources, arrange workers with the same ability as much as possible in an evaluation system, or conduct more relevant training for low-ability workers to improve their ability to the same level. It is to increase the enthusiasm of workers to invest in safety and effectively reduce the unsafe behaviors of workers.

### Design of Incentive Coefficient Based on Multiple Heterogeneity

The purpose of designing competition incentives for heterogeneous workers in construction projects is to achieve the allocation of completion goals of project and reward resources. The above analysis proves that establishing an incentive mechanism based on their ranking for heterogeneous abilities can help workers propose the optimal level of safety investment. In order to quantify the ideal tournament incentive effect, this section will calculate and discuss the incentive coefficients in the tournament incentive model. Construction projects often use linear incentive contracts to motivate participants (Fang et al., 2016). According to the HM linear incentive model, it is assumed that the principal has all the output. In order to motivate the agent, the principal must pay remuneration to the agent. And part of the reward is linked to some objective evaluation indicators (such as profit, output and product quality), then:

(14) $$W_{i}=b+\beta \,\pi_{i}$$

Among them, *b* is the fixed income paid by the owner to the construction workers; in addition, β_*i*_ is the incentive coefficient, β_*i*_ ∈ (0,1). In particular, β_1_ is the incentive coefficient of the construction worker ranked first in the competition; β_2_ = *q*β_1_ is the incentive coefficient of the second place. *q* is the decreasing incentive coefficient, 0 < *q* < 1,0 < β_2_ < β_1_.

(1) Net income of construction workers (*w*_*i*_): Under the incentive of the tournament, the actual net income of top-ranked worker is positively correlated with *W*_*i*_, positive effect of fairness preference (∂⁡Δ*W*), and negatively correlated with safety input costs *c*(*e*_*i*_).Therefore, the net income of the construction workers ranked first can be expressed as:

(15) $$\begin{array}{c} w_{i}=W_{i}-c\,\left(e_{i}\right)+\partial \left(W_{i}-W_{j}\right)\\ =b+\beta_{1}\,\pi_{i}-\frac{c\,\alpha_{i}\,e_{i}^{2}}{\lambda_{i}}+\partial \left[b+\beta_{1}\,\pi_{i}-\left(b+\beta_{2}\,\pi_{j}\right)\right]\\ =b+\beta_{1}\,\left(e_{i}+\varepsilon_{i}\right)-\frac{c\,\alpha_{i}\,e_{i}^{2}}{\lambda_{i}}+\partial \left[\beta_{1}\,\left(e_{i}+\varepsilon_{i}\right)-\beta_{2}\,\left(e_{j}+\varepsilon_{j}\right)\right]\\ =b+\beta_{1}\,e_{i}-\frac{c\,\alpha_{i}\,e_{i}^{2}}{\lambda_{i}}-\left(\partial \beta_{1}\,e_{i}-\beta_{2}\,e_{j}\right)+\beta_{1}\,\eta \end{array}$$

Among them, η = ε_*i*_ + ∂⁡(ε_*i*_−*q*ε_*j*_), η is a random variable that obeys a normal distribution. It represents the overall interference from the outside world. Since two construction workers are working on the project together, it is believed that the external interference received by the two workers is similar, thus ε∈(0,σ)2.

The actual net income of the construction workers ranked second (*w*_*j*_) is positively correlated with the incentive benefits of the manager (*W*_*j*_), and negatively correlated with the safety input cost of productive efforts *c*(*e*_*i*_) and the reverse effect of fairness preferences (δΔ*W*).Then, the net income of construction workers ranked second can be expressed as:

(16) $$\begin{array}{c} w_{j}=W_{j}-c\,\left(e_{j}\right)-\delta \,\left(W_{i}-W_{j}\right)\\ =b+\beta_{2}\,\pi_{j}-\frac{c\,\alpha_{j}\,e_{j}^{2}}{\lambda_{j}}+\delta \,\left[b+\beta_{1}\,\pi_{i}-\left(b+\beta_{2}\,\pi_{j}\right)\right]\\ =b+\beta_{2}\,\left(e_{j}+\varepsilon_{j}\right)-c\,\alpha_{j}\,e_{j}^{2}+\partial \left[\beta_{1}\,\left(e_{i}+\varepsilon_{i}\right)-\beta_{2}\,\left(e_{j}+\varepsilon_{j}\right)\right]\\ =b+\beta_{2}\,e_{j}-\frac{c\,\alpha_{j}\,e_{j}^{2}}{\lambda_{j}}-\partial \left(\beta_{1}\,e_{i}-\beta_{2}\,e_{j}\right)+\beta_{2}\,\mu \end{array}$$

Among them, μ = ε_*i*_−δ(ε_*i*_/*q*−ε_*j*_) represents the overall interference from the outside world. Therefore, μ is a random variable that obeys a normal distribution, thus ε∈(0,σ)2.

According to transaction cost economics (Liu et al., 2016), the deterministic equivalent income of the top-ranked worker ($\tilde{w_{i}}$):

(17) $$\begin{array}{c} \tilde{w_{i}}=w_{i}-\frac{1}{2}\,\rho \,V\,a\,r\,\left(w_{i}\right)=w_{i}-\frac{1}{2}\,\rho \,{\left(w_{i}-E\,w_{i}\right)}^{2}\\ =b+\beta_{1}\,e_{i}-\frac{c\,\alpha_{i}\,e_{i}^{2}}{\lambda_{i}}+\partial \left(\beta_{1}\,e_{i}-\beta_{2}\,e_{j}\right)-\frac{\rho \,\beta_{1}^{2}\,\sigma^{2}}{2} \end{array}$$

Similarly, the deterministic equivalent net income of second-ranked worker *j* is:

(18) $$\begin{array}{c} \tilde{w_{j}}=w_{j}-\frac{1}{2}\,\rho \,V\,a\,r\,\left(w_{j}\right)=w_{j}-\frac{1}{2}\,\rho \,{\left(w_{j}-E\,w_{j}\right)}^{2}\\ =b+\beta_{2}\,e_{j}-\frac{c\,\alpha_{j}\,e_{j}^{2}}{\lambda_{j}}-\delta \,\left(\beta_{1}\,e_{i}-\beta_{2}\,e_{j}\right)-\frac{\rho \,\beta_{2}^{2}\,\sigma^{2}}{2} \end{array}$$

(2) The net total benefits to the manager: According to Hypothesis 4, managers are risk-neutral (Liu et al., 2016). Therefore, the expected net income of managers is:

(19) $$\begin{array}{c} E\,U=\pi_{i}-W_{i}+\pi_{j}-W_{j}\\ =\pi_{i}-\left(b+\beta_{1}\,\pi_{i}\right)+\pi_{j}-\left(b+\beta_{2}\,\pi_{j}\right)\\ =\left(1-\beta_{1}\right)\,\pi_{i}-b+\left(1-\beta_{2}\right)\,\pi_{j}-b \end{array}$$

(3) Incentive model: Based on the classic HM principal-agent incentive model, the following constraint planning problems need to be solved when designing the tournament incentive mechanism.

For construction worker *i*, need to meet:

(20) $$\begin{array}{c} \max\left(1-\beta_{1}\right)\,\pi_{i}-b\;\left(P\,C\right)\\ b+\beta_{1}\,e_{i}-\frac{c\,\alpha_{i}\,e_{i}^{2}}{\lambda_{i}}+\partial \left(\beta_{1}\,e_{i}-\beta_{2}\,e_{j}\right)-\frac{\rho \,\beta_{1}^{2}\,\sigma^{2}}{2}\geq w_{0}\,\left(I\,R\right)\\ \max b+\beta_{1}\,e_{i}-\frac{c\,\alpha_{i}\,e_{i}^{2}}{\lambda_{i}}+\partial \left(\beta_{1}\,e_{i}-\beta_{2}\,e_{j}\right)-\frac{\rho \,\beta_{1}^{2}\,\sigma^{2}}{2}\,\left(I\,C\right) \end{array}$$

For construction worker *j*, need to meet:

(21) $$\begin{array}{c} \max\left(1-\beta_{2}\right)\,\pi_{j}-b\,\left(P\,C\right)\\ b+\beta_{2}\,e_{j}-\frac{c\,\alpha_{j}\,e_{j}^{2}}{\lambda_{j}}-\delta \,\left(\beta_{1}\,e_{i}-\beta_{2}\,e_{j}\right)-\frac{\rho \,\beta_{2}^{2}\,\sigma^{2}}{2}\geq w_{0}\,\left(I\,R\right)\\ \max b+\beta_{2}\,e_{j}-\frac{c\,\alpha_{j}\,e_{j}^{2}}{\lambda_{j}}-\delta \,\left(\beta_{1}\,e_{i}-\beta_{2}\,e_{j}\right)-\frac{\rho \,\beta_{2}^{2}\,\sigma^{2}}{2}\,\left(I\,C\right)\\ \beta_{2}=q\,\beta_{1} \end{array}$$

According to the solution method of incentive model, the optimal safety input level and incentive coefficient of the first and second construction workers are, respectively:

(22) $$e_{i}=\frac{\beta_{1}\,\lambda_{i}\,\left(1+\partial \right)}{\alpha_{i}}$$

(23) $$e_{j}=\frac{\beta_{2}\,\lambda_{j}\,\left(1+\delta \right)}{\alpha_{j}}$$

(24) $$\beta_{1}=\frac{\lambda_{i}\,\left(1+\partial \right)}{1-\partial^{2}+\alpha_{i}\,\sigma^{2}}$$

(25) $$\beta_{2}=\frac{\lambda_{j}\,\left(1+\delta \right)}{1-\delta^{2}+\alpha_{j}\,\lambda_{j}\,\sigma^{2}}$$

According to Equations (24) and (25), we obtain the incentive coefficients of the first (β_1_) and second-ranked construction worker (β_2_). In order to facilitate the analysis, this section selects two workers to establish the model. It is worth noting that this incentive model is also applicable to tournament involving multiple construction workers (*i* > 2). According to the above calculation, when there are more than two workers participating in the competition, the incentive coefficient of the third-ranked is β_3_ = *q*β_2_ = *q*^2^β_1_. Therefore, the incentive coefficient of the nth-ranked worker is β*_n_* = *q^n−1^*β_1_.

### Analysis Results

According to the results of the championship incentive model, the following analysis results:

(1) The results of salary incentives Equations (13) show that the optimal safety input level ($e_{i}^{*}$)is positively correlated with the salary gap (Δ*W*). The optimal safety investment level ($e_{i}^{*}$) increases with the increase of Δ*W* given by the manager. Conversely, according to Δ*W* = β_1_π_1_−β_2_π_2_ = β_1_(*e*_1_−*qe*_2_), it can be seen that the greater the safety input gap, the greater the ΔW. This forms a virtuous incentive cycle, and the salary incentive mechanism is an effective means to improve workers’ optimal effort. The team manager can keep the reward gap unchanged and retain the utility to minimize the total reward. That is, workers will not increase unsafe behaviors due to relaxation in this situation.
(2) According to the Equations (22) and (23), the optimal safety input $e_{i}^{*}$ of the first and second-ranked construction workers is inversely proportional to their cost coefficient of safety input (α_*i*_) and risk preference λ_*i*_. In addition, the optimal safety input ($e_{i}^{*}$) of the first-ranked worker is proportional to the reward preference coefficient when winning the first place, and the optimal safety input j of the second-ranked worker is proportional to the jealous preference coefficient when losing the first place. This indicates that the optimal safety input ($e_{i}^{*}$) is positively correlated with the fair preference coefficients ∂ and δ. Compared with the competition incentive model without considering the fair preference, the workers’ optimal effort level is improved with the consideration of the fair preference. Regardless of the rank of the workers, the optimal safety input of workers always increases with the increase of the fairness coefficient.
(3) Combining Equations (12), (22), and (23), we can study the impact of championships on workers’ unsafe behaviors under the situation of heterogeneous ability. When the worker’s cost coefficient of safety input α_*i*_ gradually increases, the degree of heterogeneity of the ability of the two workers increases, and the worker’s optimal safety input will also decrease significantly. Due to the lack of effort of the workers, the probability of accidents has greatly increased, and the championship mechanism has become inefficient. At the same time, as the degree of heterogeneity of the two workers’ abilities increases, the winning probability of worker *i* gradually increases, and the winning probability of worker *j* gradually decreases. In a practical sense, when scoring safety performance, the score of *i* is more likely to be ahead of the score of *j*. This is likely to cause the dissatisfaction of *j*and lead to a negative attitude. Therefore, for team managers, how to deploy personnel, arrange workers with the same ability as much as possible in an evaluation system, or conduct more relevant training for low-ability workers to improve to high level. It is a prerequisite for increasing the enthusiasm of workers to invest in safety behaviors and enabling the tournament mechanism effective.
(4) According to the calculation results of the incentive coefficients β_1_ and β_2_ in Equations (23) and (24), it can be calculated that the partial derivative of the fair preference coefficient ∂ to the incentive coefficient β_1_ is greater than 0. Similarly, the partial derivative of the incentive coefficient (β_2_) is also greater than 0. That is, the two incentive coefficients are both incremental functions of the fairness preference coefficient. Therefore, the higher the level of fairness preference, the greater the value of incentive coefficients β_1_ and β_2_. In addition, as the safety performance (π_*i*_ = *e*_*i*_ + ε) of construction workers increases with *e*_*i*_ and *e*_*j*_, therefore, increasing the incentive coefficient can indirectly lead to an increase in the overall safety performance of the construction project.

## Numerical Analysis

In the pre-construction stage, the manager should determine the competition incentive clauses based on the fairness preference (∂ and δ), the risk preference coefficient λ_*i*_ of each construction worker, and the influence of uncertain factors σ^2^. It is worth noting that the working abilities of these two construction workers are different, and the safety input cost coefficient of each construction worker is α_*i*_. Team managers can obtain the fairness preference (∂ and δ), and risk preference λ_*i*_ (λ_*i*_ ∈ (0,1)) of each worker through questionnaires. By judging the complexity of technology and the external natural environment, the value of uncertain factors σ^2^ ∈ (0,1) can be determined.

**FIGURE 1:**
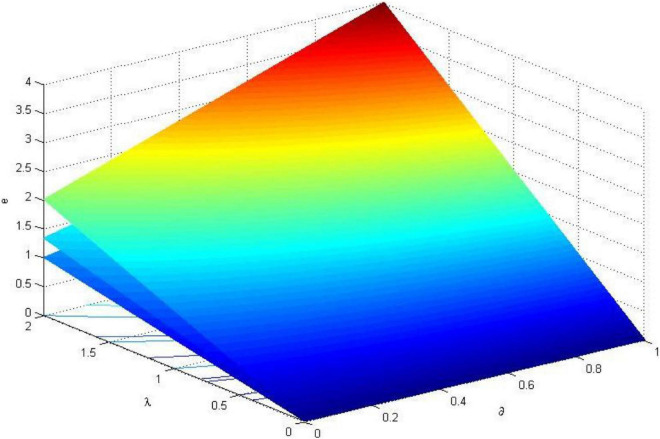
The trend of the optimal safety input level *e*_*i*_ * of construction workers (σ^2^ = 0.8).

**FIGURE 2:**
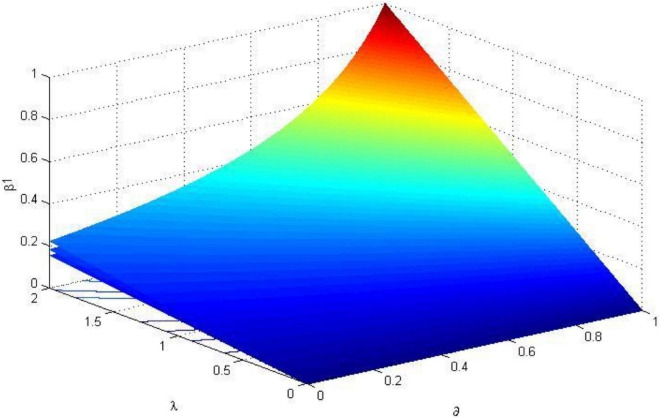
The trend of the incentive coefficient of the first-ranked worker β_1_ (σ^2^ = 0.8).

**FIGURE 3:**
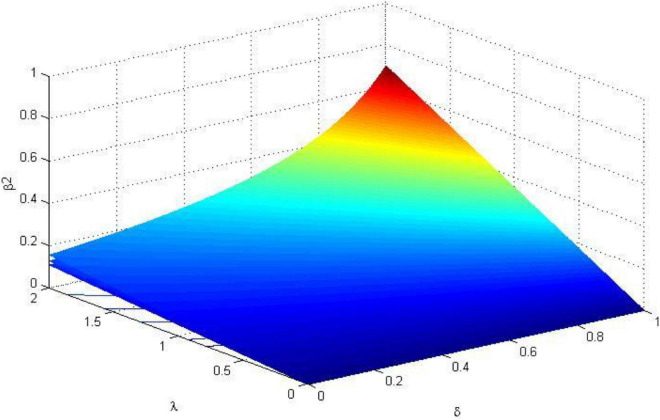
The trend of the incentive coefficient of the second-ranked worker β_2_ (σ^2^ = 0.8).

In order to formulate a reasonable incentive coefficient, the relationship between the fair preference coefficient, risk preference coefficient, safety input cost coefficient, the first and second ranked incentive coefficient are analyzed in this section. Using MATLAB to visualize the analysis results, the relationship between the parameters is shown in Figures 1–3.

In the three-dimensional coordinate system of Figure 1, the three curved surfaces from top to bottom are the levels of optimal safety investment when the cost coefficient of safety investment α_*i*_ = 1, α_*i*_ = 1.5, α_*i*_ = 2. It can be seen from the figure that when α_*i*_ increases, the optimal safety investment $e_{i}^{*}$ also increases. And the higher the worker’s ability, the greater the slope of the surface corresponding to the ability, that is, the marginal effect of α_*i*_ increases. When α_*i*_ is fixed, the optimal level of safety investment $e_{i}^{*}$ is positively correlated with the pride ∂ when winning the reward or the jealousy δ when losing the reward in the fairness preference. When 0 < λ_*i*_ < 1, that is, when the worker’s attitude is risk preference, the worker’s safety input level $e_{i}^{*}$ is generally low, and the safety input level does not change much with the increase of fairness preference; When λ_*i*_ > 1, the worker’s risk attitude is evasive, and the safety input level $e_{i}^{*}$ increases rapidly as the degree of fairness preference ∂ and λ_*i*_ strengthen. This finding shows that, regardless of whether the fairness preference is strong or not, workers with risk-averse attitudes generally have lower safety investment than workers with risk preference.

It can be seen from Figures 2, 3 that the incentive coefficient trends of the first-ranked worker *i* and the second-ranked worker *j* are consistent. In the three-dimensional coordinate system of Figures 2, 3, the three curved surfaces from top to bottom are the incentive coefficients when the safety input cost coefficient α_*i*_ = 1, α_*i*_ = 1.5, α_*i*_ = 2. In the case of α_*i*_ unchanged, when the first worker’s pride preference c increases, the incentive coefficient β_1_ increases. Since the excitation coefficient of the second place is β_2_ = *q*β_1_, (0 < *q* < 1), the excitation coefficient β_2_ also increases accordingly. When the safety input cost coefficient α_*i*_ decreases (that is, the capability of safety increases), the incentive coefficients β_1_ and β_2_ increase; When the values of ∂ and λ_*i*_ are low, the excitation coefficient increases slowly with the increase of ∂ and λ_*i*_; When ∂ > 0.6 and λ > 1, that is, only when the level of fairness preference is high and the risk attitude is evasive, the incentive coefficient increases rapidly. Therefore, managers should give higher incentive coefficients to workers who risk aversion and a high level of fairness preference.

## Discussion and Conclusion

### Discussion

The tournament incentive model designed in this paper fully considers the role of competition and the heterogeneous characteristics of multiple participating workers. It can be seen from the analysis results of the model that the tournament considering fairness preference can motivate workers to increase their safety investment. And verify the conclusions of this paper through the analysis of examples.

Tournament incentives based on the heterogeneous characteristics of workers can play the following two roles: (1) The salary gap in tournament can motivate all workers to increase safety investment during the construction process; (2) For construction workers with a higher level of fairness preference and risk aversion, the manager should formulate a larger incentive coefficient. This cannot only optimize the safety investment of each worker, but also provide more benefits for managers, thus creating a win-win situation.

When implementing the tournament incentive mechanism, it should be noted that: (1) Workers’ risk aversion attitude has a more obvious impact on their safety investment than fairness preference. When workers’ risk-averse attitudes are evasive, the remuneration given by managers can get more workers’ safety input in return. Therefore, shift managers should avoid to choose workers with a risk attitude of preference. When they have to adopt workers with a risk attitude preference, the manager should try to arrange workers with similar preferences to compete in a team. (2) In multiple rounds of repeat tournaments, workers can roughly figure out the ability level of their opponents through the previous rounds. Employees with high ability may reduce their efforts, while employees with low ability may also think that they have the low probability of winning it and gave up. As a result, tournaments can lead to inefficiency.

### Conclusion

Unsafe behavior is the most important and direct cause of accidents. Managers usually develop effective incentives to improve the safety performance of employees. Due to the large number of workers in construction projects, and these workers usually have uneven abilities and differences in various qualities, the time and energy spent by each worker in safety investment will vary to a large extent. Managers need to achieve effective and safe work incentives for heterogeneous workers through salary rewards, thereby reducing the occurrence of unsafe behaviors. By introducing the novel reward means of competition mechanism, this paper considers many psychological factors such as construction workers’ fairness preference, risk preference and ability difference, constructs the competition incentive mechanism model from the perspective of workers’ heterogeneity, and introduces it into workers’ safety behavior incentive measures. In this way, this study provides a new scheme to control the unsafe behavior of heterogeneous workers, and also gets a series of management enlightenment:

(1) Managers should pay attention to the differences in the fairness preference, risk preference and ability of construction workers, and try to choose workers with lower risk preference. The research has found that workers with risk-averse attitudes generally have a higher level of safety investment regardless of whether they have a strong preference for fairness.
(2) Managers can stimulate workers’ fairness preferences. After selecting a construction worker group, this can be achieved by designing reasonable competition contract clauses. Moreover, the reward gap is an effective way to encourage workers to invest their best efforts. On the premise of satisfying the incentive compatibility constraints of the model, managers can appropriately increase the incentive gap to achieve the best level of safety input.
(3) The different risk attitudes have the most significant impact on the safety investment level of miners participating in the tournament mechanism. The derivation of the model shows that workers whose risk attitude is evasive can bring a higher level of safety input, while workers whose risk attitude is preferred are significantly lower in safety input. The greater the difference in risk attitudes among workers, the more inefficient the incentive mechanism will be.
(4) As the degree of heterogeneity among workers increases, unsafe behaviors of workers generally increase. Therefore, for team managers, personnel and resource allocation are required. Try to arrange workers with the same ability and the same preference in one evaluation system, or provide more relevant training for workers to improve their ability to the same level. This is a prerequisite for improving workers’ safety input enthusiasm, effectively reducing the occurrence of unsafe behaviors of workers.

## Data Availability Statement

The original contributions presented in the study are included in the article/supplementary material, further inquiries can be directed to the corresponding author/s.

## Author Contributions

LJ, YZ, and WL: conceptualization. WL and YZ: methodology. LJ: software, supervision, investigation, project administration, and funding acquisition. LJ and WL: validation. WL: formal analysis, resources, data curation, writing—original draft preparation, and visualization. LJ and YZ: writing—review and editing. All authors have read and agreed to the published version of the manuscript.

## Conflict of Interest

The authors declare that the research was conducted in the absence of any commercial or financial relationships that could be construed as a potential conflict of interest. The handling editor declared a past co-authorship with one of the authors WL.

## Publisher’s Note

All claims expressed in this article are solely those of the authors and do not necessarily represent those of their affiliated organizations, or those of the publisher, the editors and the reviewers. Any product that may be evaluated in this article, or claim that may be made by its manufacturer, is not guaranteed or endorsed by the publisher.
